# Effects of variable practice on the motor learning outcomes in manual wheelchair propulsion

**DOI:** 10.1186/s12984-016-0209-7

**Published:** 2016-11-23

**Authors:** Marika T. Leving, Riemer J. K. Vegter, Sonja de Groot, Lucas H. V. van der Woude

**Affiliations:** 1 University of Groningen, University Medical Center Groningen, Center for Human Movement Sciences, Groningen, The Netherlands; 2 Amsterdam Rehabilitation Research Center | Reade, Amsterdam, The Netherlands; 3 University of Groningen, University Medical Center Groningen, Center for Rehabilitation, Groningen, The Netherlands

**Keywords:** Wheelchair mobility, Rehabilitation, Mechanical efficiency, Biomechanics, Motor exploration

## Abstract

**Background:**

Handrim wheelchair propulsion is a cyclic skill that needs to be learned during rehabilitation. It has been suggested that more variability in propulsion technique benefits the motor learning process of wheelchair propulsion.

The purpose of this study was to determine the influence of variable practice on the motor learning outcomes of wheelchair propulsion in able-bodied participants. Variable practice was introduced in the form of wheelchair basketball practice and wheelchair-skill practice. Motor learning was operationalized as improvements in mechanical efficiency and propulsion technique.

**Methods:**

Eleven Participants in the variable practice group and 12 participants in the control group performed an identical pre-test and a post-test. Pre- and post-test were performed in a wheelchair on a motor-driven treadmill (1.11 m/s) at a relative power output of 0.23 W/kg. Energy consumption and the propulsion technique variables with their respective coefficient of variation were calculated. Between the pre- and the post-test the variable practice group received 7 practice sessions. During the practice sessions participants performed one-hour of variable practice, consisting of five wheelchair-skill tasks and a 30 min wheelchair basketball game. The control group did not receive any practice between the pre- and the post-test.

**Results:**

Comparison of the pre- and the post-test showed that the variable practice group significantly improved the mechanical efficiency (4.5 ± 0.6% → 5.7 ± 0.7%) in contrast to the control group (4.5 ± 0.6% → 4.4 ± 0.5%) (group x time interaction effect *p* < 0.001).With regard to propulsion technique, both groups significantly reduced the push frequency and increased the contact angle of the hand with the handrim (within group, time effect). No significant group × time interaction effects were found for propulsion technique. With regard to propulsion variability, the variable practice group increased variability when compared to the control group (interaction effect *p* < 0.001).

**Conclusions:**

Compared to a control, variable practice, resulted in an increase in mechanical efficiency and increased variability. Interestingly, the large relative improvement in mechanical efficiency was concomitant with only moderate improvements in the propulsion technique, which were similar in the control group, suggesting that other factors besides propulsion technique contributed to the lower energy expenditure.

**Electronic supplementary material:**

The online version of this article (doi:10.1186/s12984-016-0209-7) contains supplementary material, which is available to authorized users.

## Background

Wheelchair propulsion offers mobility and independence to individuals who lost the ability to walk. In wheelchair propulsion, the ambulatory function of the legs is taken over by the arms. This type of ambulation is novel to most individuals with a permanent lower-body impairment and, therefore, has to be learned during the early stages of rehabilitation. The need for effective practice protocols that enhance the motor learning process of wheelchair propulsion is widely recognized. At the same time evidence-based guidelines are missing. The present study will evaluate a practice protocol that aims to facilitate the motor learning process of wheelchair propulsion, using variability as a key feature.

Research suggests that increased variability helps to improve motor learning by creating a flexible and adaptable biological system [1–4]. Movement variability is defined as fluctuations across repetitions during performance of a task. A higher variability is expected to increase the motor exploration, which in turn helps to find the most relevant motor solutions for a given task. Also in wheelchair propulsion, higher motor variability observed during a natural learning process (changes over time resulting from practice without feedback or instruction) appeared to be associated with better learning outcomes in terms of mechanical efficiency and propulsion technique [4, 5].

So far, motor learning in wheelchair propulsion was mostly investigated in a constrained, non-variable, laboratory environment [5, 6]. These highly internally valid experiments provided valuable information about the motor learning process in terms of mechanical efficiency (the ratio of external power output and energy expenditure) and propulsion technique, which are thought to represent the motor learning process in wheelchair propulsion [4, 6, 7]. The present study bases its hypotheses on the findings of these lab-based studies, but also partly moves away from a highly controlled experimental environment and towards a setting that resembles the environment of early clinical rehabilitation and daily life more closely.

Performance of daily tasks in a wheelchair, such as doing groceries, is highly variable and requires different skills e.g. turning, acceleration, maneuvering and interaction with obstacles. This sort of variability is not present when propelling a wheelchair on a treadmill at a steady velocity in a laboratory. A study that attempted to increase variability on a treadmill, using visual feedback on the propulsion technique variables, found improvements in propulsion technique but no improvement in mechanical efficiency [8]. Authors suggested that the addition of an extra constraint, which was visual feedback, may have compromised the optimization of the energy efficiency of wheelchair propulsion.

This finding inspired us to propose a practice protocol, which would increase variability in a different, more internal and ‘natural’ way, without an addition of feedback or instruction. While the feedback-induced variability required participants to ‘learn to be variable’, the current study aimed at facilitating ‘learning by being variable’. The participants in the present study were asked to perform tasks that require and stimulate variability, but contrary to the feedback-induced variability study [8], they were not explicitly asked to be variable. The tasks chosen for the current protocol were five isolated wheelchair skill tasks (such as a slalom or a sprint) and wheelchair basketball. Those tasks are inherently variable and do not require feedback or instruction, giving freedom to the learners to individually explore their motor solutions during uninstructed practice.

The goal of the current study was to determine the influence of variable practice on the motor learning outcomes. We assessed whether participants, who received variable practice (variable practice group), learned more than participants who did not receive any practice (control group). A no-practice control group was chosen to exclude the possibility where the repeated tests themselves could produce performance changes. We hypothesized that the variable practice group would improve both mechanical efficiency and propulsion technique during the push phase more than the control group. Mechanical efficiency is calculated as the ratio of power output and energy expenditure during steady-state submaximal cyclic exercise. Values of mechanical efficiency in hand-rim wheelchair propulsion hardly ever exceed 10% in experienced wheelchair users [9, 10]. A decrease in energy expenditure over time for a given task with a constant power output (i.e. an increase in mechanical efficiency) has been used to quantify the motor learning process [11, 12] and is suggested to be a global indicator for motor proficiency [13]. Improved propulsion technique is defined as a technique in which the contact angle of the hand with the handrim increases and push frequency decreases [4, 6, 7]. These changes are accompanied by a reduction of braking torque, meaning that wheelchair users grasp and release the handrim more fluently, and in turn save energy. Increasing variability in manual wheelchair propulsion can be achieved by varying the above mentioned propulsion technique variables in timing and magnitude by e.g. using shorter and longer pushes interchangeably or varying the frequency of pushes. We expect that variable practice will allow enhanced motor exploration which may lead to larger improvements in propulsion technique and mechanical efficiency compared to the control group. Variable practice is introduced in the form of wheelchair basketball practice and wheelchair-skill practice, which are thought to encourage motor exploration. Able-bodied participants were chosen because they are a homogenous group (similar age, lack of wheelchair experience and no comorbidities), which minimizes the inter-individual variation and allows to better isolate the effect of variable practice on the outcomes of motor learning process.

## Methods

### Participants

Eleven individuals in the variable practice group and twelve individuals in the control group participated voluntarily in the current study. The characteristics of both groups can be found in Table 1. Following detailed verbal and written information about the character of the study, all participants signed an informed consent before the onset of the study. The protocol of the study was approved by the Local Ethics Committee (Nr. ECB/2014.12.18_2), of the Center for Human Movement Sciences, University Medical Center Groningen, University of Groningen, The Netherlands. Inclusion criteria were being able-bodied, having no upper-extremity injuries and having no previous experience with manual wheelchair propulsion. Individuals were excluded when they suffered from any medical conditions that could influence the parameters measured in the study, including musculoskeletal disorders, primarily those involving the shoulder girdle or upper extremities.

**Table 1:** The characteristics (Mean ± SD) of the variable practice (*N* = 11) and the control group (*N* = 12)

Measure	Variable practice group (*N* = 11)	Control group (*N* = 12)	*P* value
Gender (males/females)	3/8	6/6	.400^a^
Age (years)	20.2 ± 2.0	20.7 ± 1.4	.507^b^
Body mass (kg)	73.2 ± 10.3	72.5 ± 9.4	.869^b^
Body height (m)	1.78 ± 0.1	1.81 ± 0.1	.456^b^

^a^2-sided *p*-value of a Fisher’s exact test

^b^2-sided *p*-value of an Independent Samples *t*-test

### Protocol

**Fig. 1:**
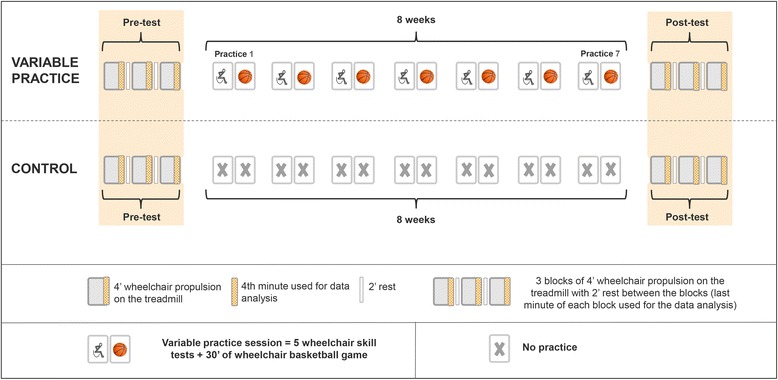
The experimental protocol for the variable practice and the control group. Both groups received identical pre and post-test. Between the pre- and the post-test, the variable practice group received 7 practice sessions, while the control group did not receive any practice

**Fig. 2:**
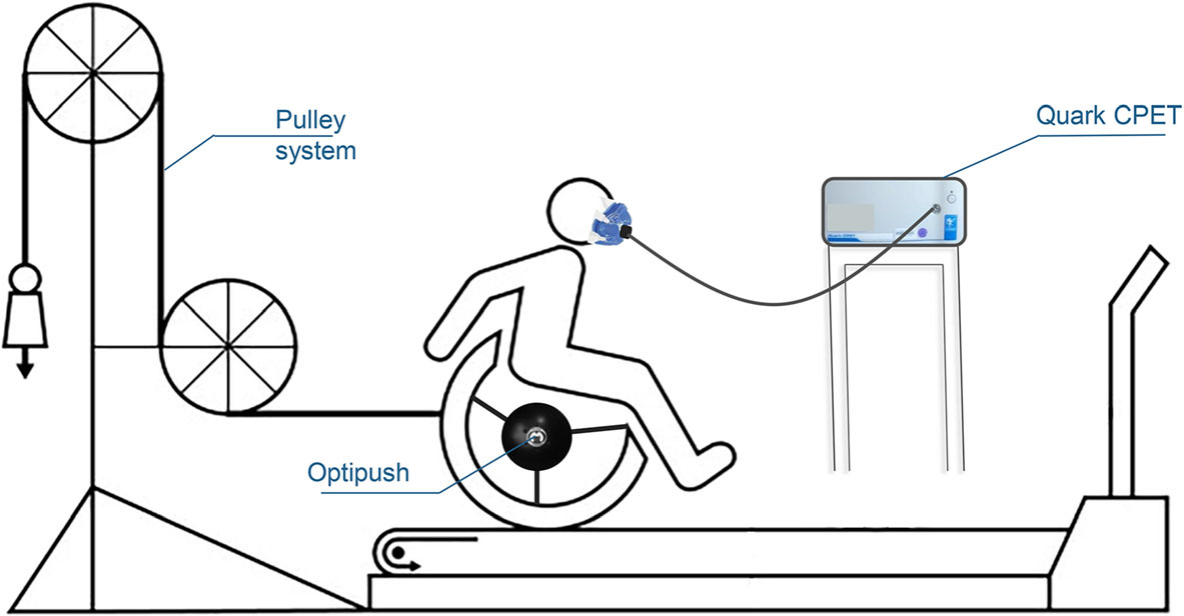
The experimental setup for the pre- and post-test. Pre- and the post-test were identical in both groups. Power output was set using the pulley system (0.23 W/kg). Treadmill speed was 1.11 m/s. The Optipush wheel measured 3D forces and torques applied to the handrim by the participant. A Quark CPET was used to determine the oxygen uptake and respiratory exchange ratio, which are necessary to calculate the mechanical efficiency. Modified figure from Vegter et al. [4]

The experimental protocol for the variable practice and the control group is presented in Fig. 1. Both groups completed the pre-test and the post-test consisting of 12 minute (3 × 4 min, with bouts of 2-min rest between the exercise blocks) wheelchair propulsion on a motor-driven treadmill under standardized conditions (Fig. 2). Time between the pre- and the post-test was the same in both groups (8 weeks). Between the pre- and the post-test the control group did not receive any intervention and the variable practice group participated in seven practice sessions. The practice sessions took place once a week. Participants who missed one session during the seven weeks (*N* = 8) received a seventh session in week 8. Each practice session lasted one hour and included the performance of five standardized wheelchair skill tasks and a 30 min uninstructed wheelchair basketball game. The actual practice time per session equaled approximately 35 min for each participant (5 min of the skill practice + 30 min of wheelchair basketball).

### Pre- and post-test protocol

Pre- and post-test in both groups were performed in the same experimental handrim Küschall K4 wheelchair (Küschall AG, Witterswil, Switzerland, 24”, no camber, seat height: 0.5 m (measured from the floor to the front of the seat), seat width: 0.47 m) placed on a 2.4 m long by 1.2 m wide level motor-driven treadmill (Forcelink b.v, Culemborg, The Netherlands). Tire pressure of the rear wheels was set at 600 kPa. Treadmill velocity was set at 1.11 m/s and power output at 0.23 W/kg body mass. The extra resistance needed to maintain the power output was calculated for each participant individually, based on the data acquired from a drag test prior to experimentation. The drag test, developed by the technical workshop of the Faculty of Human Movement Sciences at the VU University in Amsterdam, measures the rolling resistance, which together with the velocity determines the power output [14, 15]. The extra resistance was added using a pulley system [16] (Fig. 2). The experimental setup is presented in Fig. 2.

### Variable practice session protocol

#### Wheelchair skills practice

The wheelchair skill practice and wheelchair basketball game took place in a gymnasium of The University Medical Center Groningen, Centre for Rehabilitation, location Beatrixoord (Haren, The Netherlands). Eleven handrim wheelchairs were used during seven practice sessions. The five wheelchair skill tasks were performed in a wheelchair that was assigned individually to each participant for all the practice sessions, in order to make sure that possible improvement in performance between sessions was a result of the change in propulsion technique and not due to a different wheelchair. The tire pressure of the wheelchairs was standardized and checked before each practice session.

**Fig. 3:**
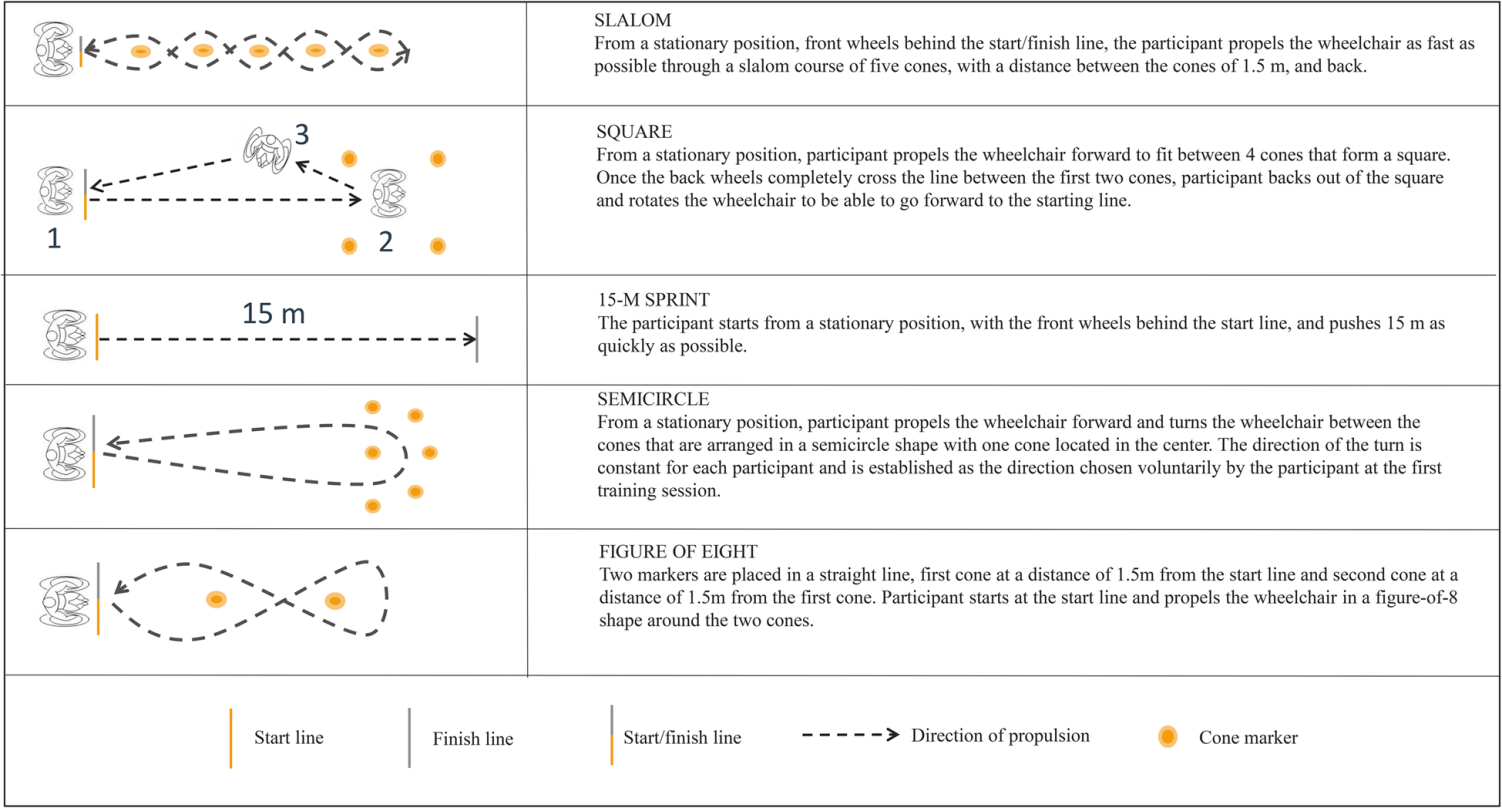
The wheelchair skill practice. Five wheelchair skill tasks were performed in the variable practice group one time by each participant at each practice session. Extra description and specifications are available in the Additional files 1, 2, 3, 4 and 5

Most important selection criterion for the skill tasks was that they had to stimulate variability and therefore involve the changes of e.g. direction, acceleration, speed. Moreover the tasks involving backward and forward handrim propulsion (represented in the present study by: slalom, figure of 8, square, semicircle), maneuvering (figure of 8 shape, slalom, square, semicircle) and sprint (15-m sprint) were used in previous research to assess the degree of wheelchair skill proficiency necessary for every day functioning [17–20]. Exact description and illustration of the tasks can be found in Fig. 3. Time for all tasks was manually recorded with a stopwatch with a precision of 0.01 s. Time was recorded from the moment the participant began to drive until the front wheels of the wheelchair passed the finish line. It took approximately 30 min to test 11 participants with the complete test battery. The sequence of the skill tasks was fixed for each participant (and counterbalanced across participants using a Latin square) to make sure that the results over time were not influenced by fatigue.

The changes within the time scores on the wheelchair skill tasks were not the main study outcome measures, but were included in the results because they give an indication about the improvement in the maneuverability across the practice sessions in the variable practice group.

#### Wheelchair basketball game

The 30-min wheelchair basketball game was performed in a different wheelchair (out of 11 available basketball wheelchairs) at every practice session in order to account for the generalization of the learned skills to different types of wheelchairs. Participants were instructed to adhere to the basic rules of wheelchair basketball as established by the International Wheelchair Basketball Federation [21]. Other than that no instructions were given.

### Data analysis

The mechanical efficiency and the propulsion technique were determined during the pre- and the post-test in both groups.

#### Mechanical efficiency

Oxygen uptake (VO2) and respiratory exchange ratio (RER) during steady-state wheelchair propulsion were continuously determined breath-by-breath using Quark Cardio-Pulmonary Exercise Testing (CPET) (COSMED, Rome, Italy). Heart rate was measured continuously throughout the experiment using CPET. The CPET was calibrated before each measurement occasion using 3 l syringe, room air and a calibration gas mixture.

Gross mechanical efficiency was calculated over the last minute of each 4-min block. The equation used to calculate mechanical efficiency was:

$$ \mathrm{M}\mathrm{E}=\mathrm{P}\mathrm{O}\times {\mathrm{E}}^{-1}\times 100\% $$

where PO is power output and E is the gross energy expenditure, calculated according to:

$$ \mathrm{P}\mathrm{O}\kern0.5em \left(\mathrm{W}\right)=\mathrm{T}\kern0.5em \left(\mathrm{torque}\kern0.5em \left(\mathrm{N}\mathrm{m}\right)\right)\times \mathrm{A}\mathrm{v}\kern0.5em \left(\mathrm{Angular}\kern0.5em \mathrm{v}\mathrm{elocity}\kern0.5em \left(\mathrm{rad}/\mathrm{s}\right)\right) $$

E(W) = (4940 × RER + 16, 040) × VO_2_ (1/min)/60, where RER and VO_2_ are the average values over the last minute of each exercise block [22].

#### Propulsion technique

The 3-dimensional forces and torques applied on the handrim of the right wheel were continuously measured during the pre- and the post-test using the software of the instrumented Optipush wheel (MAX Mobility, LLC, Antioch, TN, USA) or Smartwheel (Three Rivers Holdings, Mesa, AZ, USA). Data was sampled at 200 Hz and filtered with a fourth-order recursive Butterworth digital low-pass filter with a 20 Hz cutoff frequency [23]. The data collected during the last minute of each 4-min block were used for analysis. The output registered by the measurement wheel was calculated into specific propulsion technique variables using custom-written Matlab algorithms [4] (Table 2). The propulsion variables: frequency, braking torque and contact angle, were chosen based on their previously found association with mechanical efficiency [4]. Positive work per push and maximal torque per push were chosen as they describe the height and width of the torque signal, the properties of which the variability is likely to change as a result of variable practice. Coefficient of variation (the ratio of the standard deviation to the mean, CV = σ/μ × 100 (%)) of the five propulsion technique, separately and as an average of five propulsion variables, was used to determine the amount of variability.

**Table 2:** The propulsion technique variables. The variables were used to compare the change in propulsion technique between the pre- and the post-test. All variables, except push frequency, were calculated as an average value of all pushes performed during the last minute of each practice block. Modified table from Leving et al. [8]; Equations from Vegter et al. [4]

Propulsion variable	Unit	Description	Equation
Push frequency	push/minute	The number of pushes performed during one minute	N_pushes_/Δt
Braking torque	Nm	The braking torque applied to the handrim with each push. The sum of braking torque exerted on the handrim during coupling and decoupling of the hand	Σ_end__(i)__:start__(i + 1)_ (Tz · ΔØ)
Contact Angle	degrees (°)	The angle measured along the handrim, where subject’s hand maintained contact with the handrim during each push	Ø_end__(i)_−Ø_start__(i)_
Max Torque	Nm	The maximum torque generated around the wheel axle within a push	Max_start(i):end(i) _(Tz)
Positive Work	J	The torque around the wheel axle intergrated over the contact angle of the push	Σ_start__(i)__:end__(i)_ (Tz · ΔØ)

Abbreviations: *t*: time(s); *start(i)*: start of the current push (sample); *end(i)*: end of the current push (sample); *Tz*: torque around wheel axle (Nm); *Ø*: angle (rad)

### Statistical analysis

Statistical analysis concerning the characteristics of the participants was performed using IBM SPSS Statistics version 21.0 (SPSS Inc., Chicago, IL, USA). All data showed normal distribution at baseline, therefore parametric tests were applied. Baseline values of age, body mass and height of the participants, as well as values of all outcome measures at the pre-test, were compared between the variable practice and the control group, using independent *t*-test, to check for presence of the initial differences. Difference in the number of men and women between the groups was compared using Fisher’s exact test.

To examine the effect of variable practice on the outcomes of motor learning process, pre- and post-test values of mechanical efficiency, energy expenditure, heart rate and propulsion technique were compared between the variable practice and the control group using MLwiN version 2.31 (Center for Multilevel Modeling, University of Bristol, Bristol, UK). The data from the 3 pre-test blocks (4 min each, last minute used for the analysis) and from the 3 post-test blocks were compared. Pre- and post-test were represented in the model as time in minutes. Dummy coding was used to distinguish between the groups (0-variable practice; 1-control). Considering the possible influence of the power output on the mechanical efficiency and propulsion technique (and therefore on the variability), it was checked whether there was a difference in the power output between the pre- and the post-test between the groups (time × group effect) and within the groups (time effect). In order to prevent bias, in all cases where relative power output differed between two conditions, it was chosen to correct for it by adding power output to the model. The following regression equation was used:

$$ \begin{array}{l}\mathrm{Outcome}\kern0.5em \mathrm{measure}={\upbeta}_0\mathrm{Constant}+{\upbeta}_1\kern0.5em \mathrm{Time}\kern0.5em \mathrm{effect}\\ {}+{\upbeta}_2\kern0.5em \mathrm{Group}\kern0.5em \mathrm{effect}+{\upbeta}_3\kern0.5em \mathrm{Time}\\ {}*\mathrm{Group}\kern0.5em \mathrm{interaction}\kern0.5em \mathrm{effect}\\ {}+\left({\upbeta}_4\kern0.5em \mathrm{Relative}\kern0.5em \mathrm{Power}\kern0.5em \mathrm{Output}\right).\end{array} $$

To determine whether variable practice influences variability during steady-state propulsion on a treadmill, coefficient of variation for the (average of) five propulsion technique variables was compared between the pre- and post-test and between the groups using the same multilevel analysis as described above. The time x group effect was the outcome of interest for all analyses.

For the variable practice group, the time scores of each of five wheelchair skill tasks were compared across seven practice sessions using repeated measure ANOVA (IBM SPSS Statistics version 21.0, SPSS Inc., Chicago, IL, USA) with time (7 practice sessions) as within-subject factor. The significance level for all above-mentioned statistical procedures was set at *p* < 0.05. When a significant main effect of time was found during the ANOVA, post-hoc pairwise comparisons were performed between the seven consecutive practice sessions, for each wheelchair skill task, in order to determine the exact location of the differences. This resulted in 6 comparisons per skill task. A Bonferroni correction was applied to correct for the number of comparisons. Significance of individual dependent t-tests was therefore set at a *P* value of less than 0.05/6 = 0.008.

## Results

All participants in the variable practice (*N* = 11) and the control group (*N* = 12) completed the pre- and the post-test. Moreover all participants in the variable practice group completed 7 practice sessions. The relative power output between the pre-test and the post-test differed significantly within each group (time effect) and between groups (time × group effect) (Table 3). As a result, the relative power output was added to all multilevel regression models as a correction factor.

**Table 3:** Change in mechanical efficiency, energy expenditure, heart rate, propulsion technique and variability (CV) between the pre- and the post-test for the variable practice (*N* = 11) and the control group (*N* = 12). Mean and SD of the original data. *P* values are based on multilevel regression model outcomes (main effect of time and interaction effect time × group)

	Variable practice N = 11			Control N = 12			
	Mean^a^ ± SD			Mean^a^ ± SD			
Outcome Measure	Pre-Test	Post-Test	*p* value Time	Pre-Test	Post-Test	*p* value Time	*p* value Time × Group
Mechanical Efficiency (%)	4.5 ± 0.6	5.7 ± 0.7	<0.001	4.5 ± 0.6	4.4 ± 0.5	0.587	<0.001
Energy Expenditure (W)	368.2 ± 59.8	303.7 ± 42.6	<0.001	372.4 ± 61.0	346.7 ± 41.1	0.047	0.070
Heart rate (beats per minute)	125.9 ± 27.4	103.1 ± 19.0	<0.001	108.2 ± 21.4	100.6 ± 16.6	0.441	0.027
Propulsion Technique (unit of measurement)							
Frequency (pushes/min)	65.4 ± 12.3	57.8 ± 8.6	0.011	72.3 ± 17.2	60.0 ± 16.7	0.008	0.296
Contact Angle (degrees)	67.0 ± 8.6	77.6 ± 9.1	<0.001	57.7 ± 11.9	70.3 ± 12.8	0.001	0.322
Braking Torque (Nm)	−1.1 ± 0.8	−0.5 ± 0.4	<0.001	−1.0 ± 0.7	−0.7 ± 0.5	0.088	0.216
Positive Work (J)	9.0 ± 1.8	9.7 ± 1.8	0.369	8.3 ± 2.0	9.0 ± 2.6	0.105	0.397
Max Torque (Nm)	12.5 ± 2.2	11.6 ± 1.9	0.084	13.2 ± 2.0	12.3 ± 2.1	0.305	0.729
Variability (%)							
CV Mean^b^	21.4 ± 4.2	32.7 ± 6.1	<0.001	20.5 ± 4.6	18.5 ± 3.2	0.484	<0.001
CV Frequency	7.5 ± 3.0	6.7 ± 4.2	0.536	9.5 ± 5.1	6.8 ± 2.3	0.113	0.335
CV Contact Angle	10.7 ± 3.0	34.4 ± 5.6	<0.001	12.8 ± 3.6	9.3 ± 2.4	0.013	<0.001
CV Braking Torque	48.7 ± 18.9	70.5 ± 25.3	0.091	40.8 ± 9.9	43.6 ± 15.6	0.228	0.200
CV Positive Work	22.0 ± 4.0	38.4 ± 7.2	<0.001	23.2 ± 5.7	16.6 ± 4.2	0.002	<0.001
CV Max Torque	19.2 ± 3.7	15.4 ± 2.8	<0.001	18.5 ± 3.6	15.7 ± 3.4	0.053	0.242
Relative Power Output (W/kg)	0.22 ± 0.01	0.23 ± 0.02	0.005	0.23 ± 0.01	0.21 ± 0.02	<0.001	<0.001

^a^Mean of 3 blocks of pre- or post-test

^b^Mean CV of Frequency, Contact Angle, Braking Torque, Positive Work and Max Torque

The characteristics (age, body mass and height) of the participants were not different between the groups at baseline (Table 1). The values of all outcome measures including mechanical efficiency, propulsion technique and propulsion variability were not different between the groups at the pre-test (*p* > 0.05). The only exception was contact angle which was significantly higher in the variable practice group compared to the control group (*p* = 0.04).

### Mechanical efficiency and heart rate

**Fig. 4:**
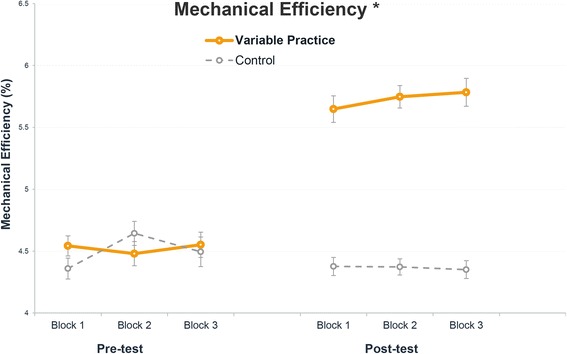
Change in mechanical efficiency between the pre- and post-test in the variable practice (*N* = 11) and the control group (*N* = 12). Mean and standard error of original data are provided per practice block. (*) Significant (*p* < 0.05) effect of time × group determined by the multilevel regression modeling

The change in mechanical efficiency of both groups between the pre- and post-test is shown in Fig. 4. As presented in Table 3, the variable practice group increased the mechanical efficiency with an absolute 1.2% (relative 27%) over the practice period (*p* < 0.001). Mechanical efficiency in the control group remained unchanged (*p* = 0.587). Moreover, the interaction effect (time × group) reached significance (*p* < 0.001), indicating that the variable practice group improved the mechanical efficiency in contrast to the control group.

Heart rate decreased significantly between the pre- and post-test in the variable practice group (*p* < 0.001) (Table 3) and remained unchanged in the control group (*p* = 0.441). Moreover, the interaction effect (time × group) reached significance (*p* < 0.027), confirming that the heart rate in the variable practice group decreased more than in the control group.

### Propulsion technique

**Fig. 5:**
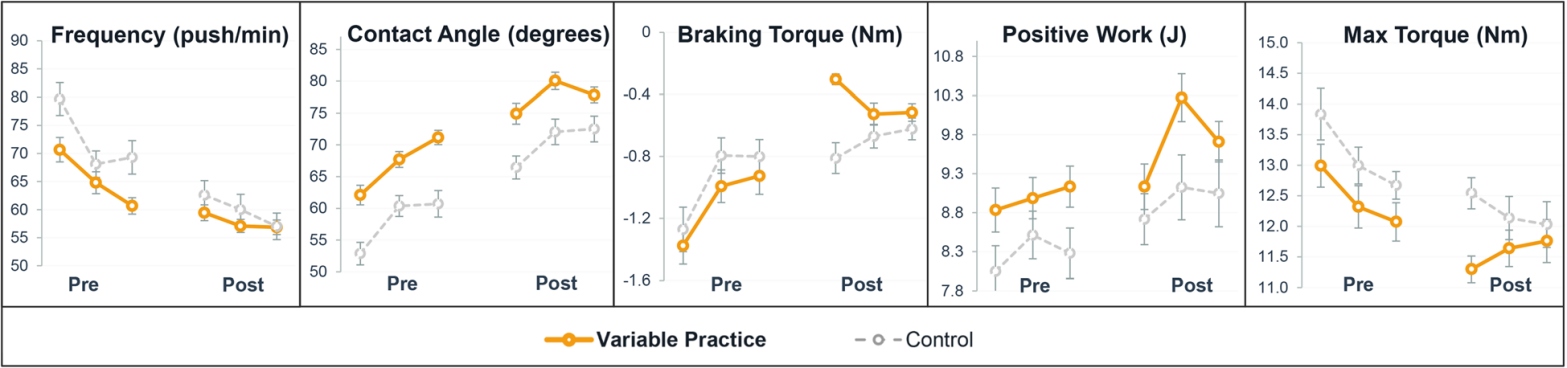
Change in propulsion technique between the pre- and post-test in the variable practice (*N* = 11) and the control group (*N* = 12). Mean and standard error of original data are provided per practice block. The time x group interaction effect was not significant for all propulsion variables

The differences in propulsion technique between the pre- and post-test are presented in Table 3 and Fig. 5. Both groups significantly reduced the push frequency and increased the contact angle of the hand with the handrim. Additionally, the variable practice group reduced the braking torque at (de) coupling. No significant changes were found in both groups for the positive work per push and max torque per push. The time x group interaction effect was not significant for all propulsion variables implying that there were no differences in the change of propulsion technique over time between groups.

### Variability

**Fig. 6:**
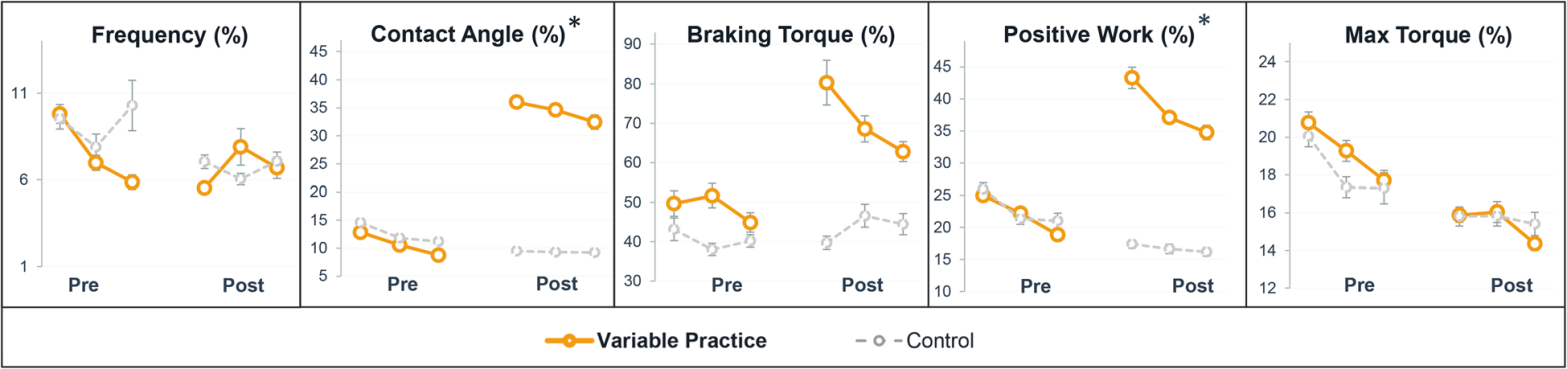
Change in variability (CV) between the pre- and post-test (%) in the variable practice (*N* = 11) and the control group (*N* = 12). Mean and standard error of original data are provided per practice block. (*) Significant (*p* < 0.05) effect of time × group determined by the multilevel regression modeling

Mean variability increased significantly between the pre and post-test in the variable practice group and did not change in the control group (Table 3). The interaction effect was significant which means that the variable practice group became more variable compared to the control group. Variability of contact angle and positive work changed in opposite direction in the two groups, i.e. it increased in the variable practice group and decreased in the control group. The time x group interaction effect for these two variables was significant. Variability of max torque decreased in both groups, although in the control group this change was not significant. There were no significant changes in the variability of frequency and braking torque in both groups. The course of variability during the pre- and the posttest is presented in Fig. 6.

### Wheelchair skill practice

**Fig. 7:**
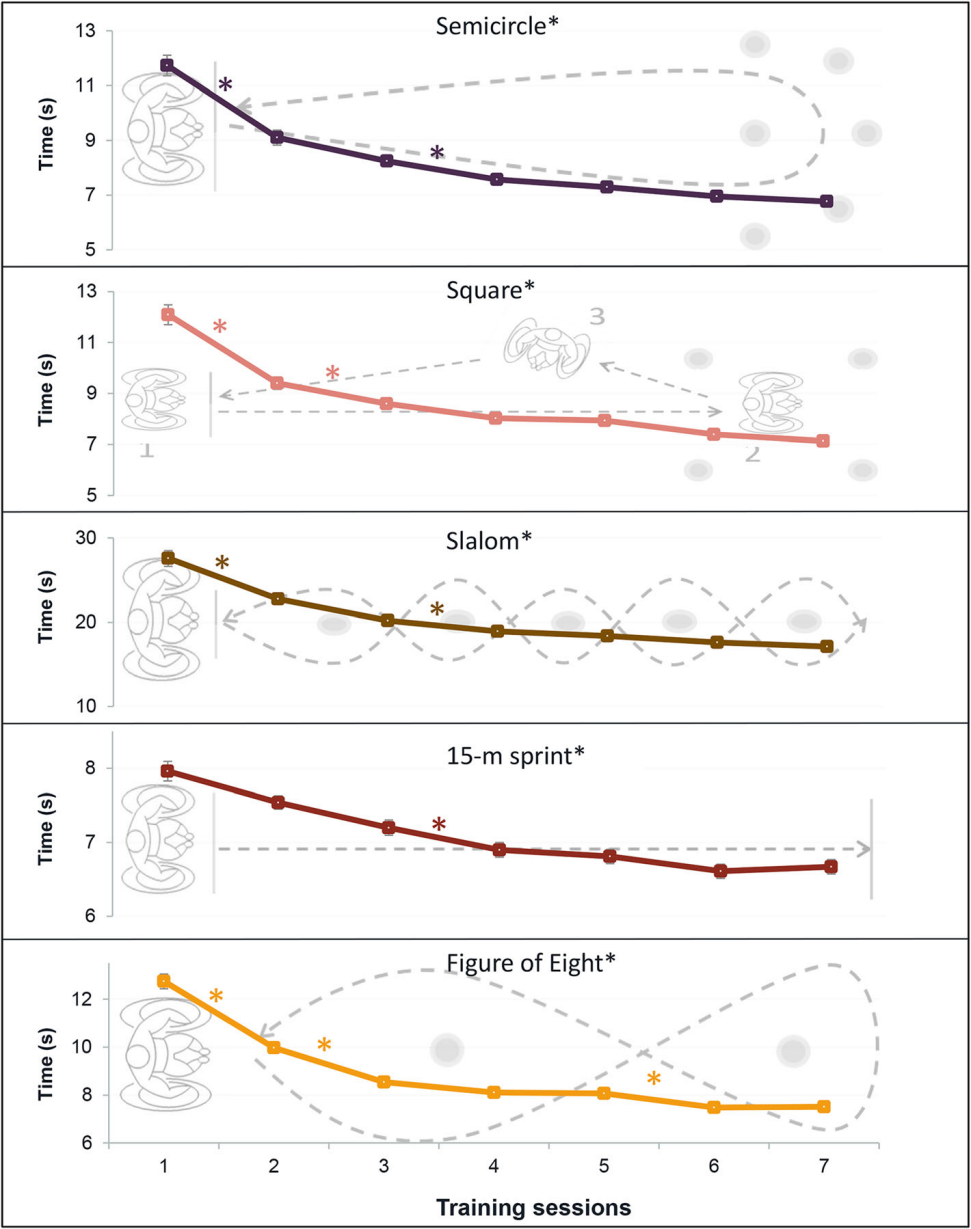
Results of repeated measure ANOVA showed that participants in the variable practice group (*N* = 11) improved their performance between the first and last session on all wheelchair skill tasks (*p* < 0.05). Mean and standard error per practice session are provided. (*) Significant (*p* < 0.008) differences between the consecutive practice sessions, determined with post-hoc tests

Participants in the variable practice group improved their performance significantly on all wheelchair skill tasks across the seven practice sessions. Figure 7 shows the exact location of the differences as determined by the post-hoc analysis.

## Discussion

The goal of the current study was to determine the influence of uninstructed variable practice on the motor learning outcomes of wheelchair propulsion. Results showed that participants in the variable practice group improved their mechanical efficiency more and became more variable than the control group. Improvements in the propulsion technique between the groups over time were comparable.

### Mechanical efficiency

The purpose of this study was to understand whether and how variable learning impacts on novel motor skill. By providing fewer constraints (participants did not receive any feedback or instruction) we aimed to allow motor exploration and therefore facilitate learning.

In Table 4 the change in mechanical efficiency observed in the present study is compared with studies concerning steady-state wheelchair propulsion on the treadmill or wheelchair ergometer. For studies where we had access to data, we performed a direct statistical comparison with the current findings of the variable practice group. All these studies were, just as the current study, performed with able-bodied participants. Mechanical efficiency in the variable practice group in the current study increased over approximately 269 min (5 min of the skill practice + 30 min of wheelchair basketball = 269 min of intermittent exercise across 10 weeks) of uninstructed practice relatively by 27% (4.5% → 5.7%). This is quite a large increase compared to a control group in which mechanical efficiency remained unchanged (4.5% → 4.4%), as well as to the other studies. Participants in a feedback-induced variability study (80 min, low-intensity, 3 weeks) decreased their mechanical efficiency between the pre- and post-test (5.25% → 5.23%) [8]. Pre- and post-test protocol in that study was similar to the present study. Natural learning protocols of various durations found an increase in mechanical efficiency. The natural learning group in the experiment of Leving et al. (80 min, low-intensity, 3 weeks) increased the mechanical efficiency by 17% between pre- and posttest (5.71% → 6.67%) [8]. Another study, which also offered 80 min of low-intensity wheelchair training (within one day or 3-weeks), found 0% (5.5% → 5.5%) of relative improvement in slower learners and 20% improvement in faster learners (4.9% → 5.9%) [5]. A study that offered two training intensities across 630 min (3× per week across 7 weeks) found a relative improvement ranging from 17 to 24% [24]. The largest relative improvement in mechanical efficiency of 30% (5.37% → 6.99%) was found following a 7-week low intensity wheelchair practice program (3× per week, 70 min = total of 1470 min) [6].

**Table 4:** The results of the present and other studies concerning the change in mechanical efficiency resulting from wheelchair practice in able-bodied individuals

Study	Group	N	Treadmill/Ergometer	Total duration (min)	Velocity m/s	Power output	Mechanical Efficiency (%)^a^;	*P* value; Current vs previous studies^b^
Present study	Variable practice	11	Testing on treadmill, practice in a gym	±269	1.11	0.23 W/kg	4.5 → 5.7 (27)	-
Present study	Control	12	Testing on treadmill, no practice	24	1.11	0.22 W/kg	4.5 → 4.4 (−2)	-
Vegter et al., 2014 [5]	Fast improvers	26	Treadmill	80	1.11	0.20 W/kg	4.9 → 5.9 (20)	x
	Slow improvers	13	Treadmill	80	1.11		5.5 → 5.5 (0)	x
De Groot et al., 2008 [6]	x	14	Testing on ergometer, Practice on treadmill	1470	1.39	20% POpeak	4.32 → 5.65 (31)	0.413
	x					40% POpeak	6.41 → 8.33 (30)	0.662
De Groot et al., 2002 [7]	x	10	Ergometer	72	1.11	0.15 W/kg	5.54 → 5.87 (6)	x
	x					0.25 W/kg	7.45 → 8.11 (9)	x
Leving et al., 2015 [8]	Feedback	17	Treadmill	80	1.11	0.24 W/kg	5.25 → 5.23 (−0.4)	<0.001
	Natural learning	15	Treadmill	80	1.11	0.25 W/kg	5.71 → 6.67 (17)	0.167
de Groot et al., 2013 [24]	Training at 30%HRR	9	Testing on ergometer, Practice on treadmill	630	1.39	20% POpeak	5.36 → 6.27 (17)	0.197
						40% POpeak	7.25 → 9.0 (24)	0.684
	Training at 70%HRR	13	Testing on ergometer, Practice on treadmill	630	1.39	20% POpeak	6.25 → 7.36 (18)	0.371
						40% POpeak	8.33 → 9.85 (18)	0.220

*Abbreviations*: *AB* able-bodied, *HRR* heart rate reserve, *POpeak*, estimated peak power output

^a^Value at the pre-test → value at the post-test (relative change over time)

^b^Relative change in mechanical efficiency (%) was compared pairwise between the present and historical studies using independent samples *t*-test. ‘x’ indicates that data from a given study were not available

The duration of exercise in the present study, 269 minutes, is longer than the duration of 80-min studies which found an increase of 0–20% in mechanical efficiency but it is also considerably shorter than the 630 min study, which found 17 to 24% improvement, or 1470 min study, which found 30% of relative improvement. As shown in Table 4, where the results of the variable practice group where statistically compared with previous literature, the relative increase in mechanical efficiency in the variable practice group is comparable to the improvements found by the historical studies, which used higher exercise doses [6, 24]. Nonetheless, it should be acknowledged that the increase in mechanical efficiency in the present study might not just be the effect of increased skill and underlying coordination, but also of improved physical capacity as response to exercise.

Next to the improvement in mechanical efficiency in the present study, also a decrease in the heart rate suggests that propulsion became less strenuous for the participants. Heart rate in the variable practice group at the post-test was on average almost 23 beats per minute slower than at the pre-test. The time x group interaction effect was also significant indicating that the decrease in heart in variable practice group was larger than in the control group. As mentioned above, it should be considered whether the reduction in heart rate could solely be attributed to changes in motor control, or whether cardio-respiratory changes have also taken place because of practice. Especially that practice sessions took place in an entertaining and social setting which could have increased participant’s motivation, involvement in practice and in turn, the physiological adaptation.

However, evidence-based recommendation of the American College of Sports Medicine states (ACSM) that 150 min of moderate exercise, or 75 min of vigorous exercise per week are necessary to improve cardiorespiratory fitness [25]. Considering the moderate intensity and intermittent character of wheelchair basketball [26–28], in order to implement the ACSM guidelines and maintain the cardiorespiratory and muscular fitness, a training frequency of 3–5 sessions per week with a duration of 20–60 min each would be required [26]. Participants in the variable practice group did not meet this exercise frequency requirement, suggesting that possible improvements in the cardio-respiratory parameters might not have been of a large influence on the mechanical efficiency. Moreover, considering the intermittent character, moderate intensity and duration of the present practice it is rather unlikely that muscle hypertrophy took place [29, 30].

A more logical assumption that could account for the improvement in mechanical efficiency in the present study is the improvement in neuromuscular coordination. Neural facilitation is thought to manifest itself already in the early stages of training [29–31]. Neuromuscular adaptation results from changes in coordination and task-specific learning that occur during learning of novel skills [29]. It can, therefore, be that if certain practice facilitates motor learning, it may also influence the rate of neuromuscular adaptation [32, 33]. The present study shows that variable practice facilitates motor learning, which perhaps may have in turn influenced neuromuscular adaptation. This may have resulted in better motor coordination and a more synchronized movement, which led to a lower energy cost of wheelchair propulsion at the post-test.

### Propulsion technique

Participants in both groups improved the propulsion technique between the pre- and post-test to a similar extent. In Table 5 the change in propulsion technique observed in the present study is compared with able-bodied participant studies concerning steady-state wheelchair propulsion on the treadmill or wheelchair ergometer. The baseline values of all propulsion technique variables in both groups were similar to those found in other studies [4, 6–8]. The direction of improvement of frequency, contact angle, braking torque and positive work is in line with natural learning studies [4, 6–8]. The relative improvement is smaller in the variable practice group compared to the natural learning studies for push frequency (12 vs 22–33% respectively) and positive work (8 vs. 24–78%) and similar for contact angle (16 vs. 12–41%) and braking torque (59 vs. 48–66%). The relative improvement is smaller in the present study compared to the feedback-induced variability experiment for contact angle (16 vs 33% respectively) and push frequency (12 vs. 33%) and larger for braking torque (59 vs 13%) [8].

**Table 5:** The results of the present and other studies concerning the change in propulsion technique variables resulting from wheelchair practice in able-bodied individuals

Study	Group	Frequency (push/min)^a^	Contact Angle (degrees)^a^	Braking Torque (Nm)^a^	Max Torque (Nm)^a^	Positive Work (J)^a^
Present study	Variable practice	65.4 → 57.8 (12)	67 → 77.6 (16)	−1.1 →−0.5 (59)	12.5 → 11.6 (7)	9.0 → 9.7 (8)
Present study	Control	72.3 → 60.0 (17)	57.7 → 70.3 (21)	−1.0 →−0.7 (30)	13.2 → 12.3 (7)	8.3 → 9.0 (8)
Vegter et al., 2014 [5]	Fast improvers	68 → 49 (28)	61 → 76.2 (25)	x	12.6 → 13.0 (−3)	8.3 → 10.8 (31)
	Slow improvers	67 → 52 (22)	62.5 → 69.7 (12)	x	12.0 → 12.8 (−7)	8.1 → 10.0 (24)
De Groot et al., 2008 [6]	x	x	57.6 → 81.2 (41)	x	x	12.7 → 22.6 (78)
	x	x	66.2 → 88.5 (34)	x	x	23.8 → 36.2 (52)
De Groot et al., 2002 [7]	x	61.0 → 41.7 (32)	x	x	x	13.9 → 21.6 (56)
	x	62.8 → 46.4 (26)	x	−5.6 →−2.9 (48)	x	22.6 → 32.7 (45)
Leving et al., 2015 [8]	Feedback	62.1 → 41.5 (33)	66.3 → 88 (33)	−0.8 →−0.7 (13)	x	x
	Natural learning	71.3 → 52.5 (26)	60 → 77.5 (29)	−0.6 →−0.2 (66)	x	x

^a^Value at the pre-test → value at the post-test (relative change over time)

The direction of change in max torque in both groups in the present study was opposite to the 80-min experiment of Vegter et al. [5]. Max torque decreased (although not significantly) in the present study and increased significantly in the experiment of Vegter et al. [5]. Considering that baseline values of max torque were very similar, the difference at post-test may be caused by the lower push frequency in the study of Vegter (52 (Vegter et al.) vs. 58 (variable practice group) and 60 (control group) pushes/min), since the value of contact angle at the post-test was also very similar between the studies.

Improvement in the propulsion technique in the present study was rather moderate when compared to the relatively large improvement in the mechanical efficiency. A natural learning study suggested that improvement in mechanical efficiency is related to the improvement in the propulsion technique [4]. The relatively large improvement in the mechanical efficiency in the present study cannot completely be accounted for by the improvement in propulsion technique as captured on the level of force production on the handrim, since it was quite small compared to other studies. This suggests that, next to the currently investigated propulsion technique variables, other factors contributed to the lower energy expenditure. The upper body has redundant degrees of freedom to perform the propulsion task. The applied force comes from a combination of forces generated by the trunk, shoulder, elbow and wrist muscles. It was shown that on the short term participants transfer force production away from the elbow towards the shoulder [34]. Future studies might look at the effect of motor learning not only on measurement wheel based propulsion technique measures, but also incorporate the upper body kinematics to better understand propulsion technique changes.

Another possibly not captured change is the participant’s control over the wheelchair. The wheelchair skill tasks all showed clear improvements in performance times suggesting an improved maneuverability and control. Possibly this translated to less speed fluctuations and left-right steering correction on the treadmill, subsequently leading to less energy loss and thus a higher mechanical efficiency. The left-right steering and subsequently medio-lateral position on the treadmill has, to our knowledge, not been investigated yet in wheelchair propulsion. It could potentially be evaluated in future studies as an outcome measure of the motor learning process. The left-right steering could be seen as an equivalent of medio-lateral displacement during gait, a measure used to describe dynamic balance and ability to manifest obstacles [35–38]. Investigating it could be accomplished with the use of motion capture systems.

### Variability

Both, present study protocol for the variable practice group and natural learning protocols [4, 6–8, 24] led to improvement in mechanical efficiency and propulsion technique. The main difference between them is the course of variability, which increased in the variable practice group and either did not change or decreased in the natural learning protocols [5, 8], including the current control group. It was a surprising finding since wheelchair propulsion on a treadmill does not particularly require variability. It is not certain whether the level of variability reached in the current study is a desirable feature. From the motor exploration point of view, once a solution for a task has been found, high variability becomes superfluous. However variability may have a different role in developing a novel skill (increasing motor exploration) and different role in a formed, skilled behavior (facilitating flexibility in dealing with perturbations). Change in variability in the present study is certainly worth mentioning since the direction of change is opposite to the one during natural learning [5, 8]. However, whether increasing and maintaining variability should be a training goal for any motor skill, including wheelchair propulsion, is a subject for future research.

Increase in variability in the present study in the variable practice group was concomitant with the improvement in mechanical efficiency. This suggests that the conclusion made by Leving et al. [8] that higher variability requires more energy and therefore may disturb the energy efficiency optimization may be true for feedback-induced variability but is not in this study. The present study showed that an increase in variability can be achieved by offering variable uninstructed practice and that this increase seems to benefit the mechanical efficiency.

### Wheelchair skill practice

Participants in the present study showed improvement on all five wheelchair skill tasks. Inspection of Fig. 7 allows to see that most rapid improvement can be seen between the first and third practice session. After the fourth session, performance improvements are smaller. This seems to suggest a large short-term improvement, which is followed by some smaller long-term improvement. Most important selection criterion for the wheelchair skill tasks was that they stimulated variability during practice. This goal was achieved since participants increased the variability at the post-test. However, the improvement over the practice sessions is important in itself since good performance on wheelchair skills allows the wheelchair users to improve their life-space mobility and participation [39–43]. Moreover, it was shown that the tasks similar to the ones used here, involving backward and forward propulsion, maneuvering and sprint, can be used to assess the degree of wheelchair skill proficiency necessary for every day functioning, also during and after rehabilitation [17–20].

### Advantages and limitations

The advantage of the present study is its ecological validity and good feasibility. Facilities and equipment used during the practice sessions are present in many rehabilitation centers. Additionally, the used protocol allows training relatively large groups of participants during one hour practice sessions with minimal staff supervision. This decreases the financial, time and transportation constraints, which are commonly mentioned as barriers to physical activity in individuals with spinal cord injury [44–50].

It should be noted that while in other wheelchair practice studies, the training was performed in the same or similar conditions as the testing conditions, the practice sessions in the present study were performed in a very different setting. The fact that participants were not used to propelling on the treadmill may have influenced their energy efficiency, which may have potentially been even higher. The present study showed that propulsion technique, efficiency and variability trained in an ecologically valid setting transferred to the steady-state treadmill propulsion setting.

We think that choosing a no practice group in combination with the statistical comparison with previous literature on the influence of less variable practice on the outcomes of motor learning provides sufficient evidence in favor of variable practice in wheelchair propulsion and points to possible directions in future research. The inclusion of able-bodied subjects in this study may be seen as limitation. Selecting able-bodied participants with similar age and lack of wheelchair experience eliminates potential confounders, which are often present in the wheelchair-dependent population: e.g. lack of sitting balance, presence of pain or secondary medical complications. The inclusion of able-bodied participants ensures a homogenous group, which allows to more accurately isolate the effect of variable practice on the outcomes of motor learning process. Translation of results from this study, for implementation in clinical rehabilitation, should be done with caution. It may be that actual wheelchair users have an inhibited trunk or upper extremity function or sitting balance, which in turn could not only decrease the overall range of motion but also influence the motor variability.

The experimental wheelchair for the pre- and the post-tests, as well as the basketball wheelchairs used during the practice sessions in the variable practice group did not allow any correction for individual height or width. This could be seen as a limitation of this study. However, importantly all dimensions within the subjects were constant over the pre- and the post-test.

The exact dose of variability for each participant during the practice sessions is unknown. This is a limitation of the current study, since some participants may have been more active or more variable than others. However, when looking at the intra-individual change between the pre and the post-test in the variable practice group, we could see that all participants improved their mechanical efficiency. This would suggest that the level of activity and variability during the practice sessions was comparable between them since we could not see differences in the outcome measures. Furthermore, a researcher was always present during the practice sessions and there were no striking differences in the activity or propulsion strategy between the participants. Future studies on motor learning could benefit from task-specific activity monitors and more detailed information on wheelchair speed (preferably power output) and physical strain (i.e. heart rate) during the practice sessions.

### Future research

A recent study showed that positive changes in mechanical efficiency and propulsion technique during very early stages of motor learning process are not necessarily accompanied by a decrease in shoulder load, which gives an important indication about the injury risk [34]. Other studies point out that there may be a relationship between variability and shoulder pain [51, 52]. Future studies should evaluate whether the increase in variability, next to enhancing the motor learning process, decreases the shoulder load during manual wheelchair propulsion.

The present study provides support for the suggestion by Ranganathan and Newell [53] that various kinds of variability may influence the outcomes of motor learning process differently. Variability introduced in the present study, contrary to the visual feedback-induced variability [8], allowed for improvements in mechanical efficiency. Future research should attempt to explore the differences between various kinds of variability and their influence on the motor learning process. Moreover translation of the motor learning principles to clinical rehabilitation is important, since all novel wheelchair users go through a process of motor learning where most rapid changes happen at the beginning. Better monitoring of this process and development of evidence-based protocols are expected to positively influence the outcomes of rehabilitation.

## Conclusions

The present study showed that encouraging intrinsic variability, by introducing variable practice, resulted in an increase in mechanical efficiency and increased variability compared to a control group. Large relative improvement in mechanical efficiency was concomitant with moderate improvement in the propulsion technique suggesting that factors other than propulsion technique as measured by the instrumented wheel contributed to the lower energy expenditure. It may be that variable practice stimulated variation of propulsion technique and facilitated the exploitation of the dynamics of the task and improved coordination and/or maneuverability. This may have contributed to more efficient and thus less straining propulsion. Future research should determine whether changes in variability and the motor learning process found in the present study influence the load on the shoulder and thus injury risk resulting from wheelchair propulsion.

## Additional files

Additional file 1: The specifications of wheelchair skill task: slalom. (TIF 167 kb)
Additional file 2: The specifications of wheelchair skill task: semicircle. (TIF 154 kb)
Additional file 3: The specifications of wheelchair skill task: figure of eight. (TIF 157 kb)
Additional file 4: The specifications of wheelchair skill task: square. (TIF 258 kb)
Additional file 5: The specifications of wheelchair skill task: 15-m sprint. (TIF 142 kb)
Additional file 6: Complete data set. (XLS 106 kb)

## Abbreviations

ACSM: American College of Sports Medicine
Av: Angular velocity
CPET: Cardio-Pulmonary Exercise Testing
E: Energy expenditure
ME: Mechanical Efficiency
PO: Power Output
RER: Respiratory Exchange Ratio
T: Torque
VO2: Oxygen uptake
