# Supplementary figures and images for: Effects of variable practice on the motor learning outcomes in manual wheelchair propulsion
Source: J Neuroeng Rehabil. 2016 Nov 23;13:100. doi: 10.1186/s12984-016-0209-7 (PMC5120477; doi:10.1186/s12984-016-0209-7)

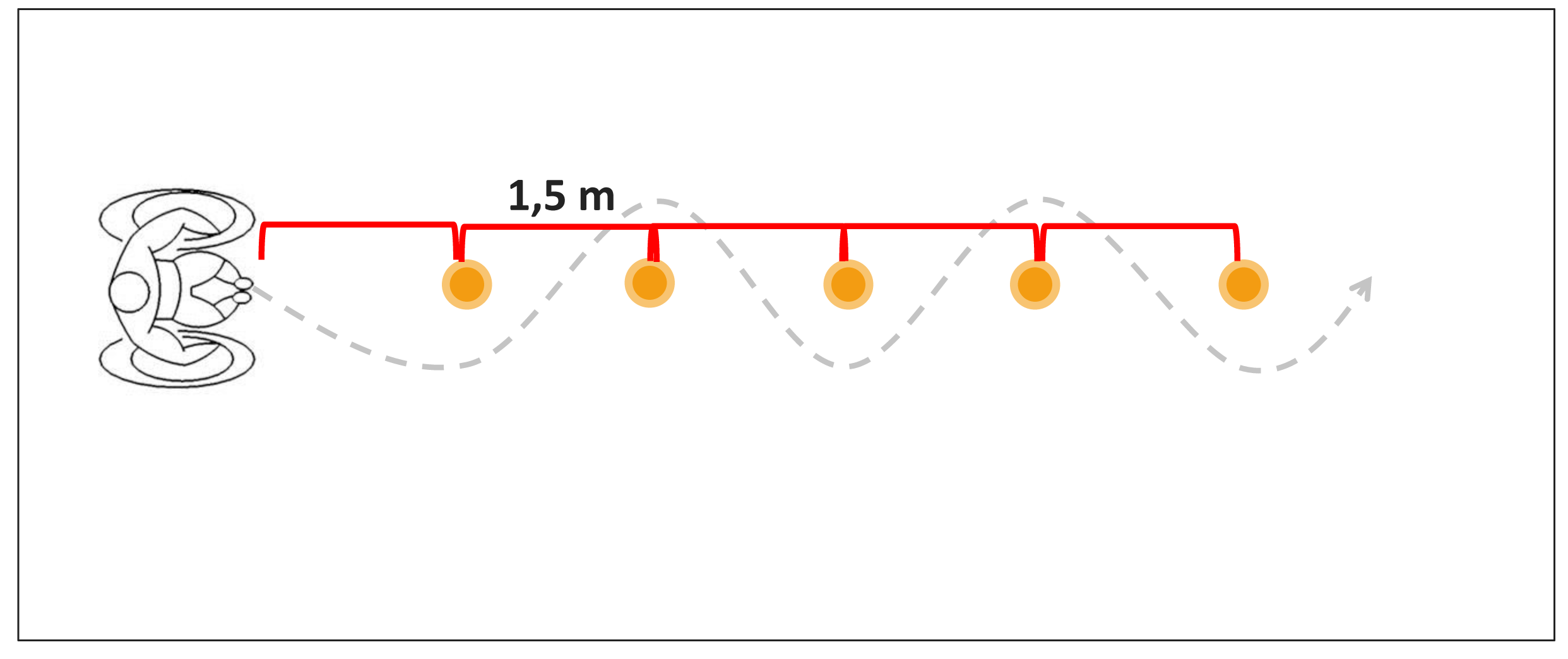

Supplement: Additional file 1: — The specifications of wheelchair skill task: slalom. (TIF 167 kb) [file 12984_2016_209_MOESM1_ESM.tif]

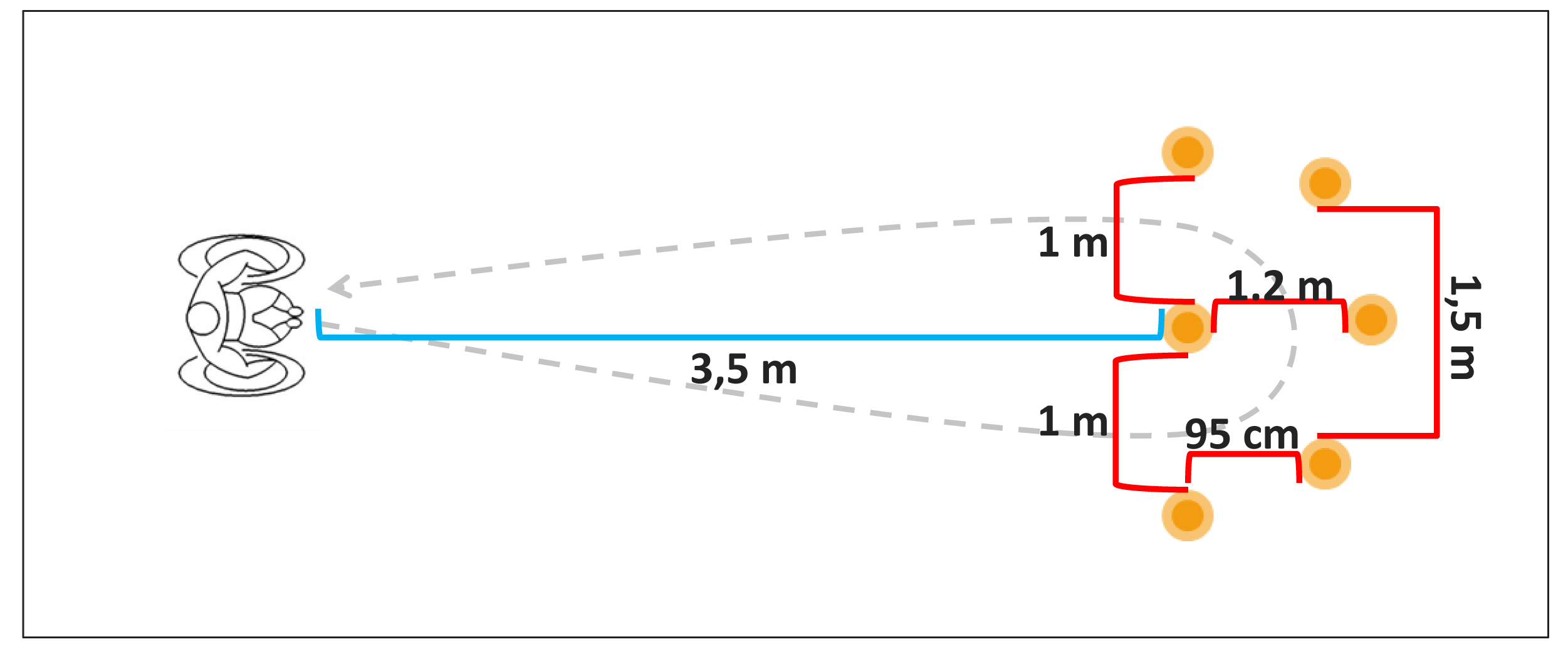

Supplement: Additional file 2: — The specifications of wheelchair skill task: semicircle. (TIF 154 kb) [file 12984_2016_209_MOESM2_ESM.tif]

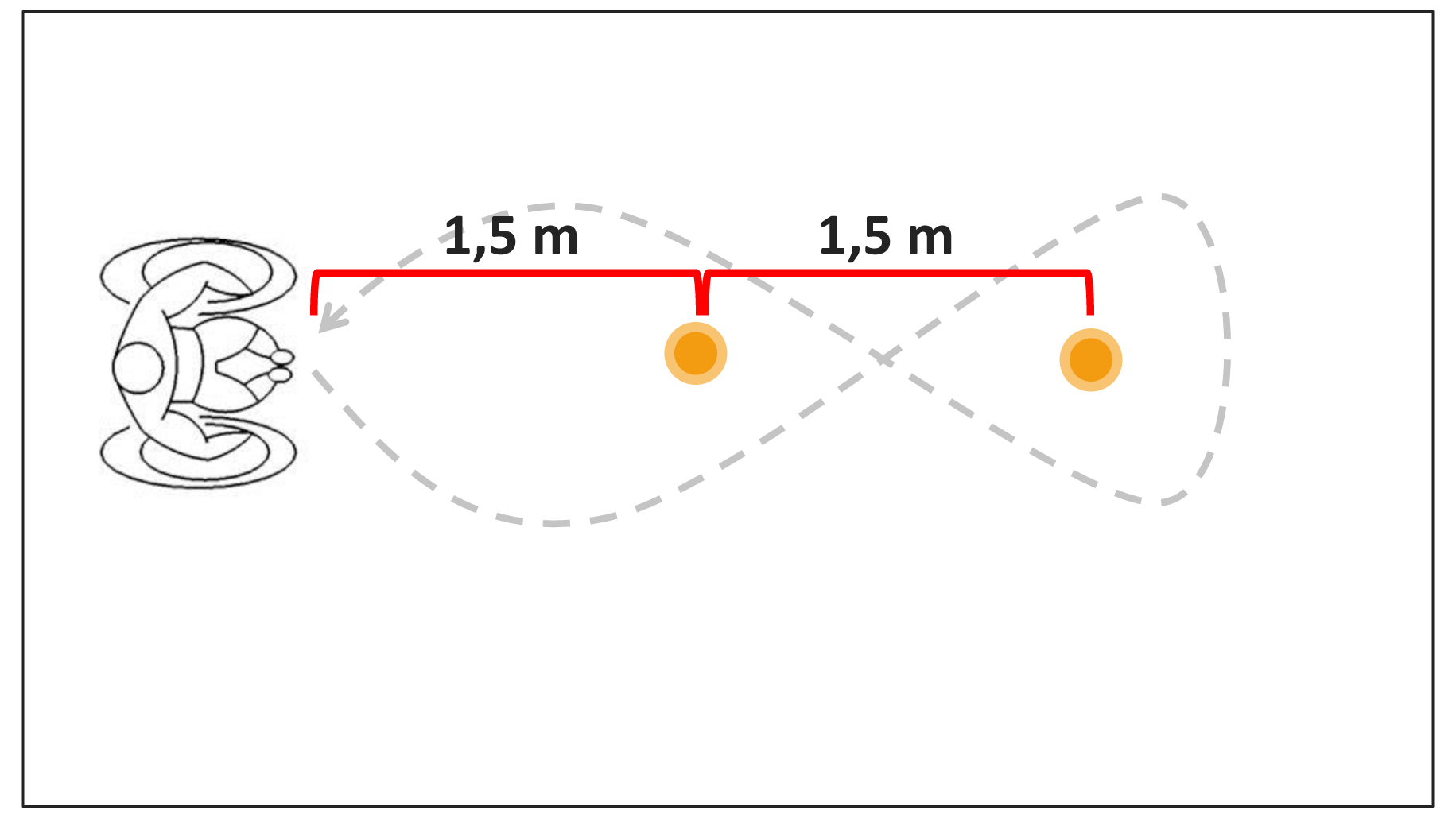

Supplement: Additional file 3: — The specifications of wheelchair skill task: figure of eight. (TIF 157 kb) [file 12984_2016_209_MOESM3_ESM.tif]

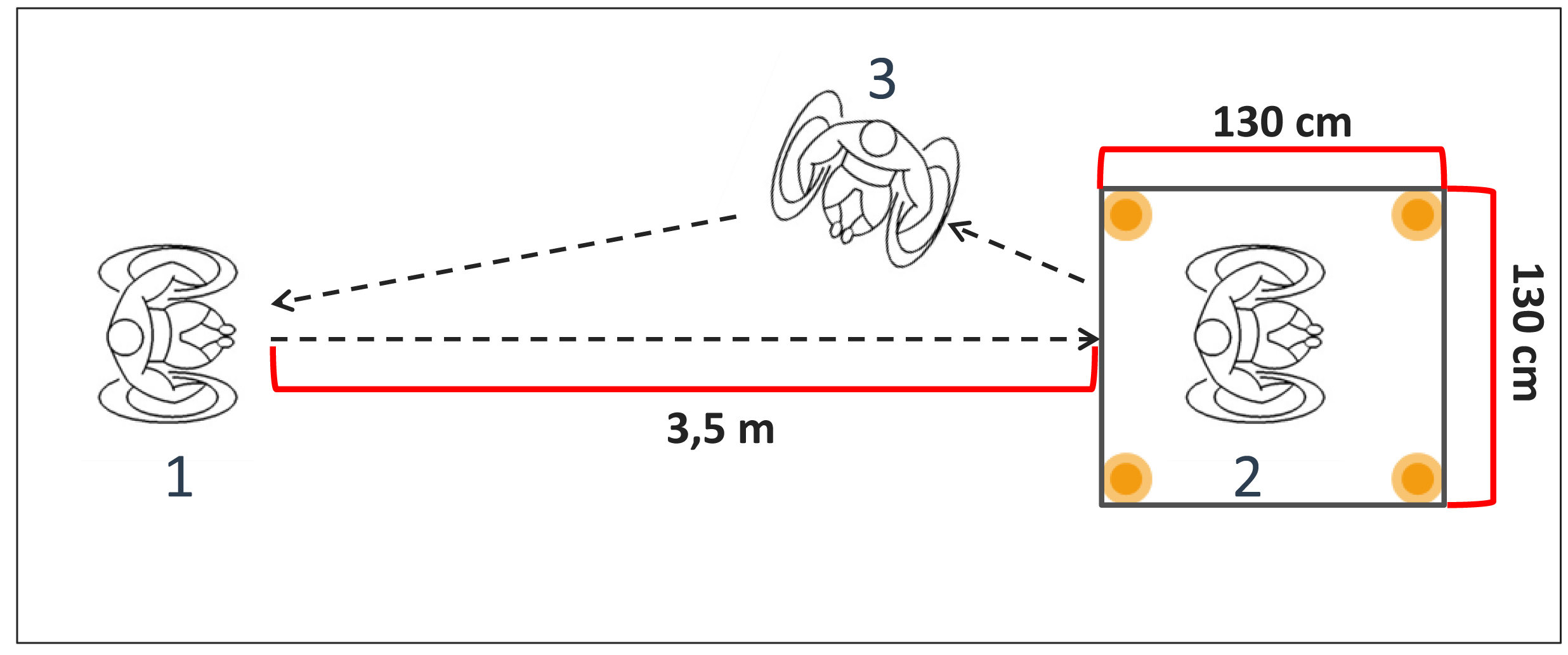

Supplement: Additional file 4: — The specifications of wheelchair skill task: square. (TIF 258 kb) [file 12984_2016_209_MOESM4_ESM.tif]

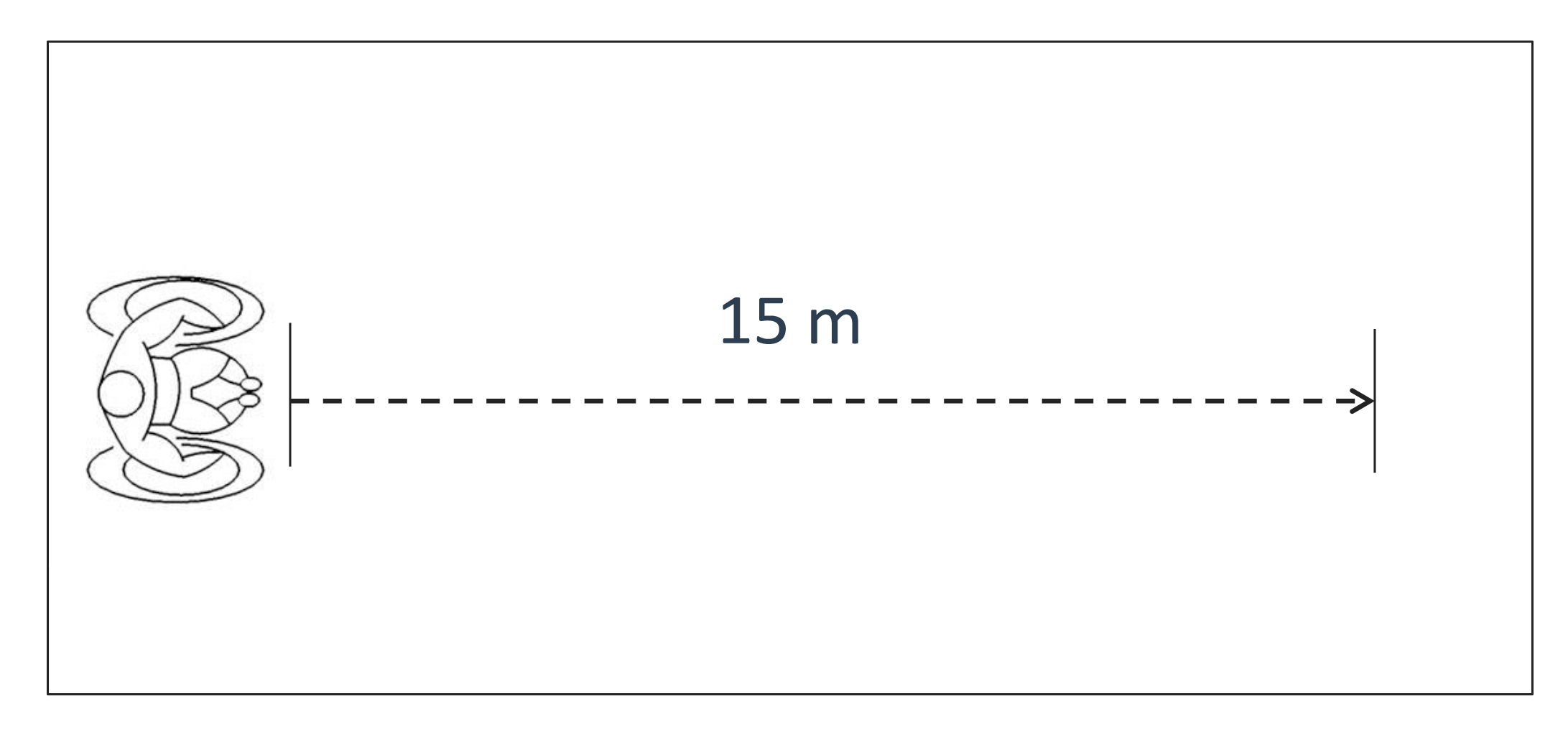

Supplement: Additional file 5: — The specifications of wheelchair skill task: 15-m sprint. (TIF 142 kb) [file 12984_2016_209_MOESM5_ESM.tif]
